# Effect of acupuncture Baihui acupoint-Yintang acupoint-Neiguan acupoint combined with conventional Western medicine treatment on cerebral blood flow in insomnia patients: a 3D-ASL functional magnetic resonance imaging study

**DOI:** 10.3389/fneur.2026.1679885

**Published:** 2026-01-22

**Authors:** Wei Wang, Xihua Lv, Shichuan Xie, Teng Jiang, Jie Zhou

**Affiliations:** 1 Department of Radiology, Shenzhen Hospital of Beijing University of Chinese Medicine (Long Gang), Shenzhen, Guangdong, China; 2 Department of Acupuncture, Shenzhen Hospital of Beijing University of Chinese Medicine (Long Gang), Shenzhen, Guangdong, China

**Keywords:** acupoint, acupuncture, curative effect, functional magnetic resonance imaging, insomnia

## Abstract

**Aim:**

To explore the effect of acupuncture Baihui acupoint-Yintang acupoint-Neiguan acupoint combined with conventional Western medicine treatment on insomnia patients by functional magnetic resonance imaging.

**Methods:**

This study adopted a randomized controlled design. From June 2021 to December 2022, 100 patients with insomnia who were admitted to our hospital were selected and divided into the control group and the study group. The control group received conventional Western medicine treatment. Based on the alprazolam treatment in the control group, the study group received acupuncture treatment. All patients underwent functional magnetic resonance imaging (fMRI) scanning. The fMRI data, sleep quality and anxiety symptoms of patients were compared between the two groups.

**Results:**

After 28 days of treatment, the average cerebral blood flow values of cerebellar cortex, thalamic nucleus, occipital lobe, frontal lobe, and parietal lobe of the study group were significantly higher than those of the control group (*p* < 0.05). After 28 days of treatment, the PSQI score and HAMA score of the study group were significantly lower than those of the control group (*p* < 0.05).

**Conclusion:**

Acupuncture Baihui acupoint-Yintang acupoint-Neiguan acupoint combined with conventional Western medicine treatment can effectively improve the sleep quality and anxiety symptoms of insomnia patients, and fMRI visualizes the effect of acupuncture, provides guidance for acupuncture treatment.

## Introduction

Insomnia is a common disorder characterized by difficulty in maintaining and/or maintaining sleep, accompanied by irritability or fatigue during wakefulness, which affects the quality of life during the day (1). It has been reported that insomnia is one of the most widespread mental disorders, with an incidence of about 10, and 35 to 50% of adults in the world have insomnia symptoms every year, which seriously affects the physical health and quality of life of patients (2). Long-term severe insomnia can cause physical and mental symptoms and increase the risk of mental and cardiovascular diseases and accidents (3). In addition, insomnia is also a risk factor for other diseases (4). At present, Western medicine mainly uses sedative and hypnotic drugs for the treatment of insomnia, but long-term use of such drugs has certain addiction, easy to develop drug resistance, and will also appear fatigue, sleep behavior disorder, ataxia, and other risks (5).

Acupuncture is the essence of traditional medicine in China and a precious cultural heritage of the Chinese nation (6). It has been the basis of clinical practice for thousands of years in China (7). It has the advantages of wide adaptability, remarkable curative effect, convenient use, economic security and so on, and has made an indelible contribution to the life and health of the Chinese nation and even the people of the world (8). A number of current studies have shown that acupuncture points have a clear effect on the treatment of insomnia (9). Due to the lack of direct theoretical support of clinical comparative studies, the physiological mechanism of acupuncture has not been fully clarified, and the principles of acupuncture cannot be explained by modern medical theories, many people are still skeptical about the exact curative effect of acupuncture (10).

In recent years, with the maturity of neuroimaging technology, it is possible to study acupuncture in human body, which opens up a new way to explore the mechanism of acupuncture (11). Functional magnetic resonance imaging (fMRI) has become a hot spot in the study of brain functional activity, and acupuncture of a certain acupuncture point on the body surface can cause changes in fMRI functional imaging, which can directly show the positioning of acupuncture effect in the center, so as to conduct real-time and intuitive research on the physiological and pathological changes of the human body in the process of clinical acupuncture, and realize functional visualization, making fMRI a method to study the effect of acupuncture on central nervous system activity, and providing an effective way for the mechanism of acupuncture therapy and the study of integrated traditional Chinese and Western medicine (12, 13).

In this study, we aimed to explore the effect of acupuncture Baihui acupoint-Yintang acupoint-Neiguan acupoint combined with conventional Western medicine treatment on insomnia patients by fMRI.

## Methods

### Study design

**Figure 1:**
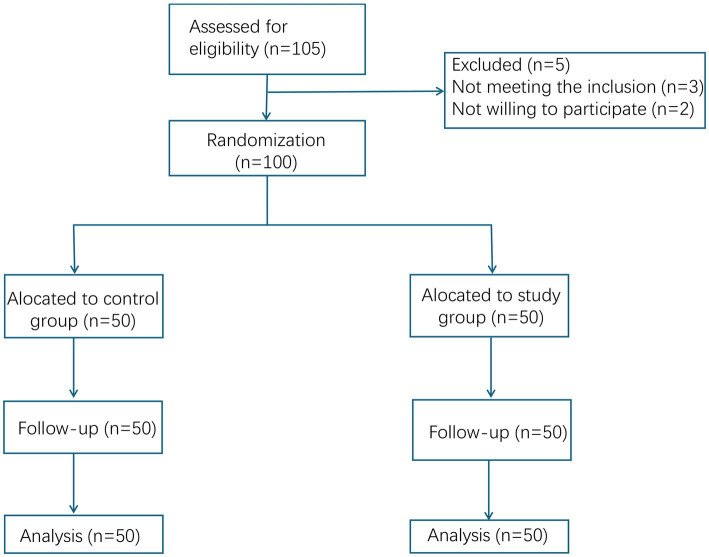
CONSORT flow diagram of participant enrollment, allocation, follow-up, and analysis.

This was an open-label trial without sham acupuncture. This study adopted a randomized controlled design. From June 2021 to December 2022, 100 patients with insomnia who were admitted to our hospital were selected as the research subjects. The flow diagram was shown in Figure 1. This study was approved by the Ethics Committee of Shenzhen Hospital of Beijing University of Chinese Medicine (Long Gang). All patients volunteered to participate in this study and signed the informed consent.

Inclusion criteria: (1) Met the diagnostic criteria for primary insomnia, no depression, anxiety and other mental or psychological disorders; (2) Pittsburgh Sleep Quality Index (PSQI) score >7; (3) Age > 18 years old; (4) No serious skin or blood-related diseases, such as neurodermatitis and atopic dermatitis, thrombocytopenia, coagulation disorders and hemophilia. Exclusion criteria: (1) Severe organic diseases such as liver and kidney dysfunction; (2) Unable to accept the treatment in this study, fainting and afraid of acupuncture, unable to cooperate with doctors to complete data collection before and after treatment; (3) There were contraindications of magnetic resonance (MR) scanning.

### Sample size calculation

Referring to the previous trial on acupuncture for primary insomnia (14), we determined that a sample size of 40 in each group would provide a power of 80% and an alpha level of 0.05 allowing us to detect the difference in PSQI score between the two groups. Allowing a 20% dropout rate, a sample size of 50 in each group was needed in this trial.

### Randomization

This study used the random number table method for random allocation. First, 100 patients who met the criteria were numbered from 1 to 100 in the order of enrollment. The starting point was selected from the random number table (e.g., the 3rd row and 4th column), and the reading direction was from right to left. Two-digit numbers were read successively, 01–50 corresponding to patients in the study group, 51–100 in the control group. Numbers beyond the range or repetitions were skipped. In the end, there were 50 patients in each group.

To ensure the confidentiality of the allocation, personnel who were not involved in the implementation of the study were arranged to put the group information into opaque sealed envelopes, with only the patient numbers marked on the envelopes. When patients were enrolled in the study, they would open the corresponding envelopes and be grouped according to the information inside the envelopes, ensuring that the researchers and the patients did not know the groupings before the grouping process.

### Blinding

To mitigate the impact of potential bias to some extent, we took measures to blind the outcome assessors. The individuals responsible for collecting and analyzing the fMRI data, as well as those scoring the PSQI and Hamilton Anxiety Scale (HAMA), were not involved in the treatment allocation process and were unaware of which group each patient belonged to.

## Methods

Patients in the control group was given conventional Western medicine treatment. They took alprazolam (manufacturing company: Hunan Dongting Pharmaceutical Co., Ltd.) orally, with a dosage of 0.5–0.8 mg each time, 30 min before going to bed, for a total of 28 days.

Based on the alprazolam treatment in the control group, the study group received acupuncture treatment. We employed electric acupuncture using the Yingdi pulse acupuncture therapy instrument. The electric acupuncture was set to deliver a continuous-wave stimulation with a frequency precisely set at 2 Hz. The intensity was adjusted to a level that elicited a mild but noticeable deqi sensation (a characteristic sensation of acupuncture, often described as a dull ache, numbness, or tingling) in the patients without causing any discomfort or pain, and this intensity level was maintained consistently throughout each 30-min treatment session. Patients received the treatment once a day for a total of 28 days.

All acupuncturists involved in this study were licensed practitioners with a minimum of 5 years of clinical experience in acupuncture. They had undergone formal training in traditional Chinese medicine acupuncture techniques and had also received specific training on the use of acupuncture therapy instrument. Prior to the start of the study, all acupuncturists participated in a standardized training session to ensure uniform understanding and implementation of the acupuncture protocol. This training included hands-on practice, theoretical explanations of the acupoint selection rationale, and detailed instructions on the operation of the electric acupuncture device. Regular quality-control meetings were held during the study period to monitor and maintain the consistency of the acupuncture operations among different acupuncturists.

### fMRI scanning

All patients underwent 1.5 T superconducting magnetic resonance scanning using a Siemens scanner (Germany). The scanning sequence employed was 3D-arterial spin labeling (3D-ASL). The scanning parameters were set as follows: repetition time (TR) was 4,500 ms, echo time (TE) was 10 ms, the matrix size was 512 × 512, the field-of-view (FOV) was 25 mm × 25 mm, the slice thickness was 4.0 mm, and the number of excitations was 3. After labeling, the delay time was 1,500 ms, and 30 layers of continuous scanning were carried out to cover the whole brain. Regarding the ASL labeling scheme, a pulsed arterial spin-labeling (PASL) approach was used, where arterial blood water is magnetically labeled by a short radiofrequency pulse applied to a selected slice in the neck region, and then the labeled blood travels to the brain for imaging.

### fMRI image analysis

The image realignment was conducted using a rigid-body transformation algorithm to correct for head motion artifacts in the fMRI data. Data with excessive head motion were excluded from further analysis. Excessive head motion was defined as exceeding 2 mm of translation in any of the three axes (x, y, z) or 2 degrees of rotation around any of the three axes (pitch, roll, yaw).

The realigned images were spatially normalized to the Montreal Neurological Institute (MNI) template using a non-linear registration algorithm to transform individual brain images into a common anatomical space.

After normalization, the images were smoothed with a Gaussian filter having a full-width at half-maximum (FWHM) of 6 mm to improve the signal-to-noise ratio and increase statistical power.

The test sites were pre-defined as the cerebellar cortex, thalamic nucleus, occipital lobe, frontal lobe, and parietal lobe. Based on the standard brain atlas, we precisely identified the boundaries of these regions in the MNI space. For each patient’s normalized and smoothed fMRI data, we extracted the corresponding voxels within these pre-defined ROIs.

The Functool-ASL graphics processing software was used to calculate the average cerebral blood flow (CBF) value on the left and right sides of each ROI. The CBF values obtained in this study are absolute values, with the unit of milliliters of blood per 100 grams of brain tissue per minute (mL/100 g/min). The software utilized the 3D-ASL data to generate CBF maps, and then we averaged the CBF values of all voxels within each ROI to obtain the mean CBF for further statistical analysis.

### Observation indicators

1. fMRI data including the average CBF value of cerebellar cortex, thalamic nucleus, occipital lobe, frontal lobe and parietal lobe.
2. PSQI (15) was adopted to assess the sleep quality of patients, and was divided into 7 dimensions, including daytime disturbance, sleep duration, sleep quality, sleep time, sleep efficiency, sleep medication and sleep disorder. The total score was 21 points, and the scoring standard for each dimension was 0 to 3 points. The higher the score, the worse the sleep quality of patients was.
3. HAMA (16) score was used to assess the anxiety level of patients, including 14 items such as anxious mood, tension, fear, and insomnia. The score is <7, indicating no anxiety symptoms; ≥7 points indicate anxiety, and the higher the score, the more serious the anxiety of the patient.

### Statistical analysis

SPSS 20.0 statistical software was adopted for statistical analysis of the data. The Shapiro–Wilk test was used to measure the normality of the data distribution for each variable. For measurement data that met the assumption of normality, we used the independent-samples t-test for comparisons between two groups. The measurement data were expressed as mean ± standard deviation (x ± s). Effect size was displayed as 95% confidence interval (95% CI). Statistical data, which were presented as frequencies and percentages [*n* (%)], were analyzed using the chi-square (*χ*^2^) test. In addition, a two-way repeated-measures analysis of variance using a Bonferroni correction for multiple comparisons was used to evaluate the level of biomarkers in plasma between the two groups. *p* < 0.05 was considered statistically significant.

## Results

### Baseline data of patients between the two groups

The baseline demographic and clinical characteristics of the patients are summarized in Table 1. As shown in Table 1, there were no significant differences in baseline data such as gender, age, BMI, course of disease and underlying disease between the two groups (*p* > 0.05).

**Table 1:** Baseline data of patients between the two groups.

Items	Control group (*n* = 50)	Study group (*n* = 50)	*χ*^2^/*t*	*p*
Gender			0.04	0.84
Male	24 (48.00)	25 (50.00)		
Female	26 (52.00)	25 (50.00)		
Age (years)	46.9 ± 1.4	46.8 ± 1.6	0.33	0.74
BMI (kg/m^2^)	23.6 ± 1.5	23.7 ± 1.3	0.35	0.72
Course of disease (months)	6.8 ± 1.5	6.7 ± 1.6	0.32	0.75
Underlying disease				
Diabetes	13 (26.00)	10 (20.00)	0.50	0.47
Hypertension	9 (18.00)	11 (22.00)	0.25	0.61

### Comparison of fMRI data between the two groups

**Figure 2:**
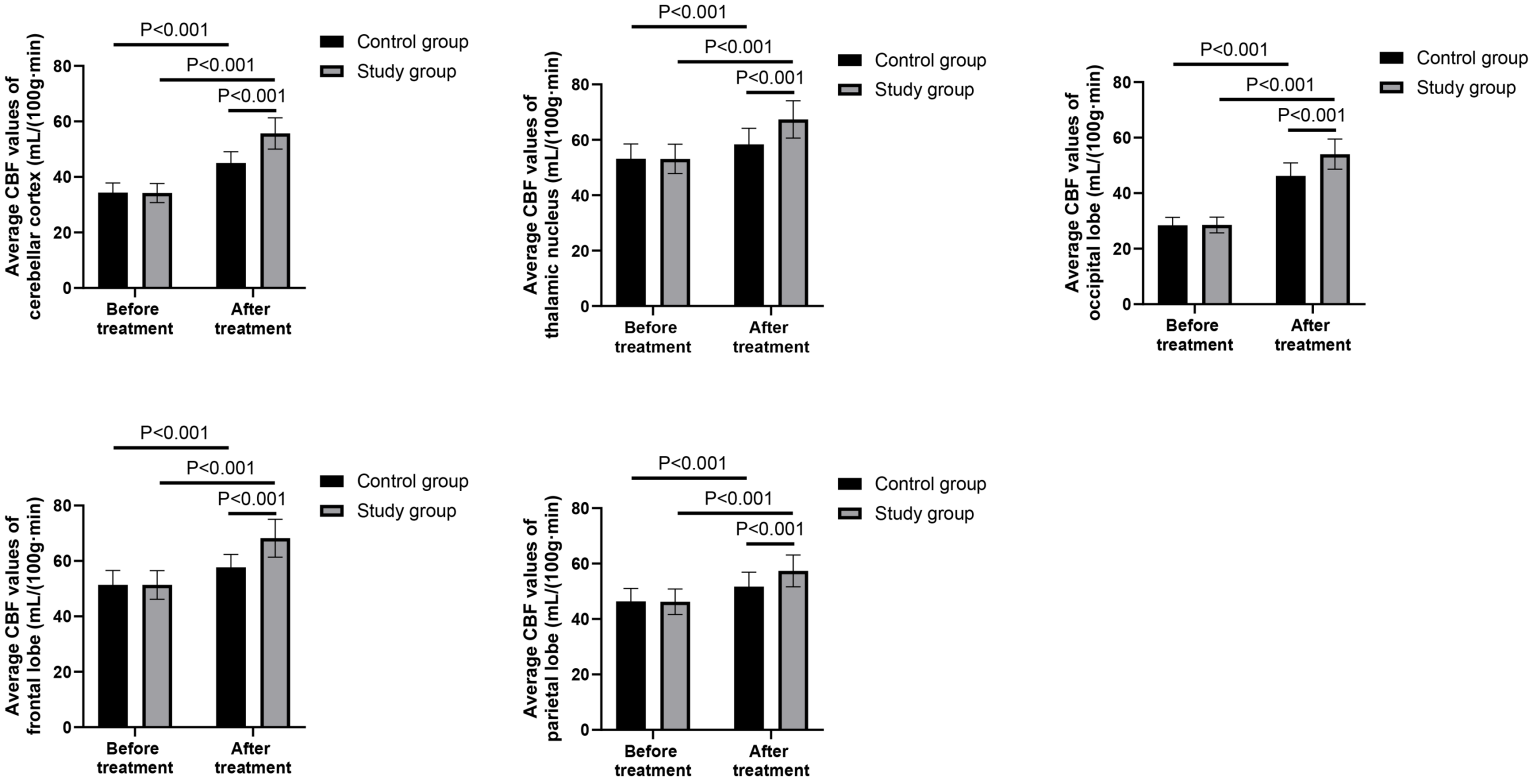
Comparison of the average cerebral blood flow (CBF) values in predefined brain regions between the two groups before and after treatment.

Before treatment, there were no significant differences in the average CBF values of cerebellar cortex, thalamic nucleus, occipital lobe, frontal lobe, and parietal lobe between the two groups (*p* > 0.05). After 28 days of treatment, the average CBF values of cerebellar cortex, thalamic nucleus, occipital lobe, frontal lobe, and parietal lobe of both groups were higher than those before treatment (*p* < 0.001, 95% CI: −6.433 to −4.067; *p* < 0.001, 95% CI: −6.102 to −2.848; *p* < 0.001, 95% CI: −5.105 to −2.815; *p* < 0.001, 95% CI: −6.779 to −3.701; *p* < 0.001, 95% CI: −4.175 to −1.345). Compared with the control group, the study group had higher average CBF values of cerebellar cortex, thalamic nucleus, occipital lobe, frontal lobe, and parietal lobe after 28 days of treatment (*p* < 0.001, 95% CI: −17.25 to −14.89; *p* < 0.001, 95% CI: −11.34 to −8.088; *p* < 0.001, 95% CI: −22.82 to −20.52; *p* < 0.001, 95% CI: −13.12 to −10.04; *p* < 0.001, 95% CI: −9.625 to −6.795; Figure 2).

### Comparison of PSQI score between the two groups

**Figure 3:**
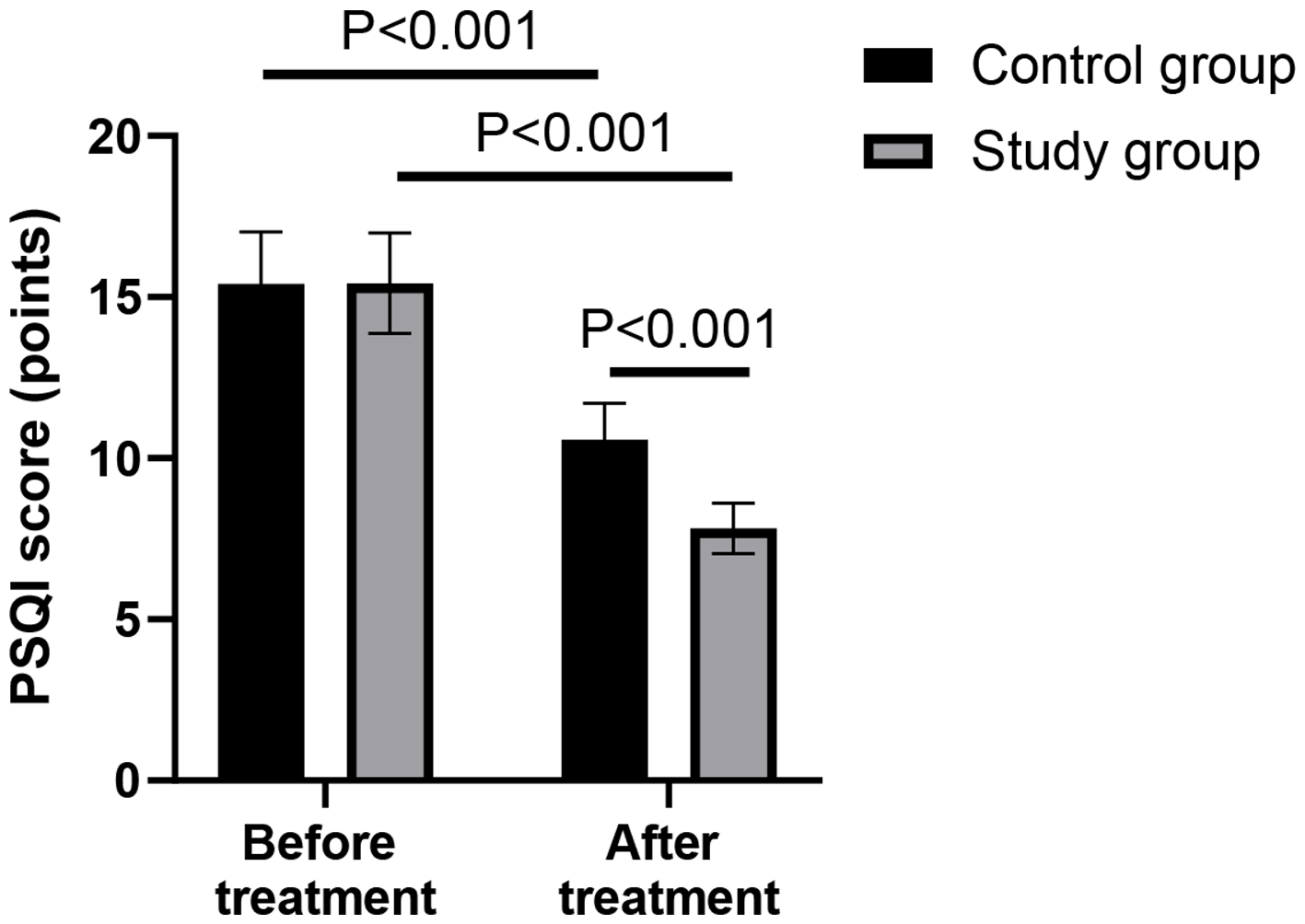
Comparison of the Pittsburgh Sleep Quality Index (PSQI) scores between the two groups before and after treatment.

Before treatment, there was no significant difference in the PSQI score between the two groups (*p* > 0.05). After 28 days of treatment, the PSQI scores of both groups were lower than those before treatment (*p* < 0.001, 95% CI: 0.998–1.732). Compared with the control group, the study group had lower PSQI score after 28 days of treatment (*p* < 0.001, 95% CI: 5.848–6.582; Figure 3).

### Comparison of HAMA score between the two groups

**Figure 4:**
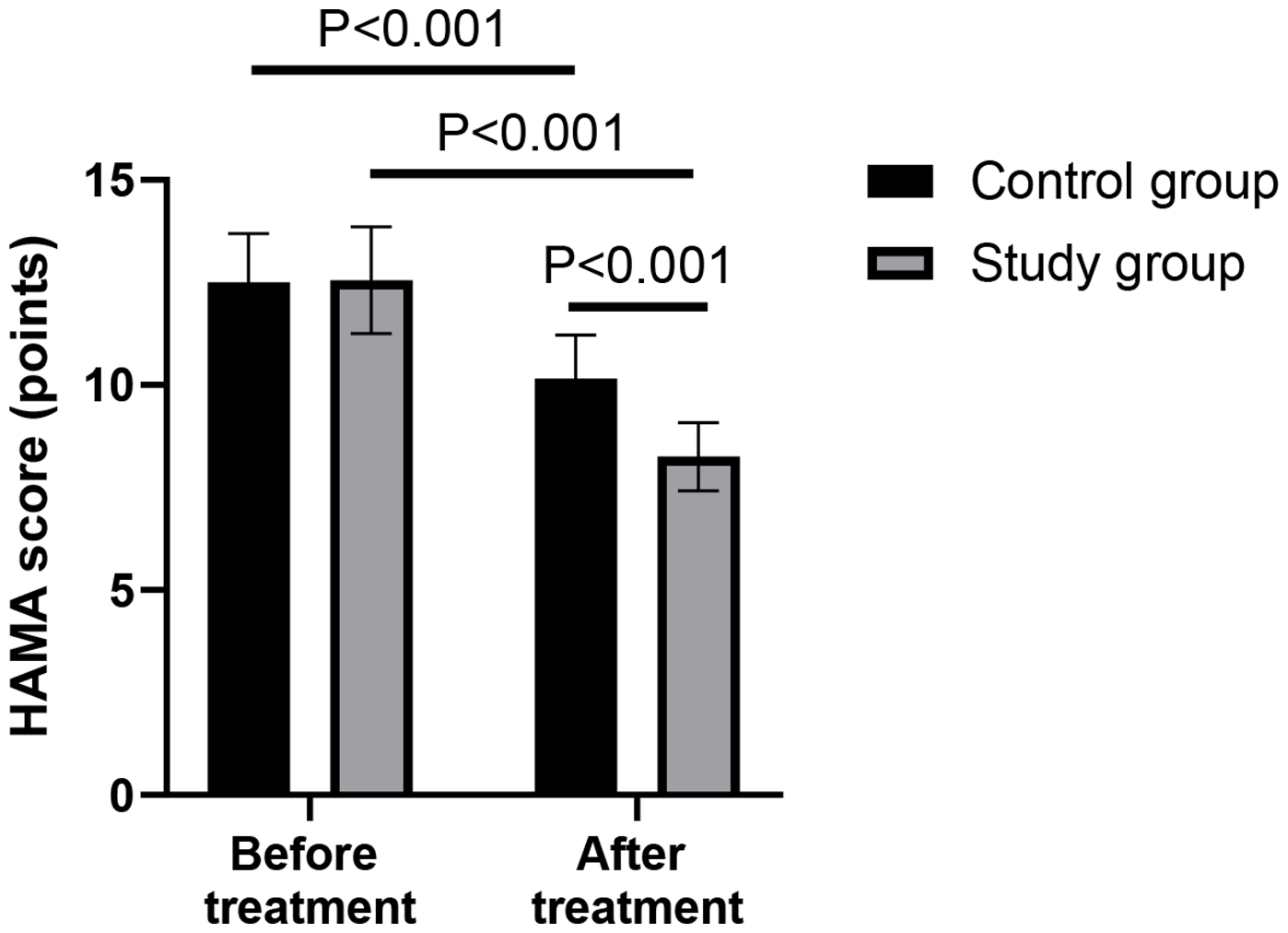
Comparison of the Hamilton Anxiety Scale (HAMA) scores between the two groups before and after treatment.

Before treatment, there was no significant difference in the HAMA score between the two groups (*p* > 0.05). After 28 days of treatment, the HAMA scores of both groups were lower than those before treatment (*p* < 0.001, 95% CI: 0.609–1.231). Compared with the control group, the study group had lower HAMA score after 28 days of treatment (*p* < 0.001, 95% CI: 3.019–3.641; Figure 4).

## Discussion

### Main findings

In our study, after 28 days of treatment, the average CBF values in the cerebellar cortex, thalamic nucleus, occipital lobe, frontal lobe, and parietal lobe of the study group were significantly higher than those of the control group. Additionally, the PSQI score and the HAMA score of the study group were significantly lower than those of the control group. These results suggest that the combined treatment can effectively improve the sleep quality and anxiety symptoms of insomnia patients, and the fMRI findings provide visual evidence of acupuncture’s effects on brain function.

However, it should be noted that the assumption that increased CBF in all ROIs simply indicates “improvement” needs to be carefully considered in the context of insomnia as a hyperarousal disorder. Insomnia is often characterized by an overactive central nervous system, and abnormal neural activity patterns are prevalent. While increased CBF in some regions may indeed be associated with improved sleep and reduced anxiety, this may not be a straightforward relationship across all brain areas (17).

### Comparison with previous literature

Previous neuroimaging studies have provided valuable insights into the neural mechanisms underlying insomnia and the potential effects of acupuncture. A study published in Heliyon in 2023 (18) investigated the neural correlates of insomnia using resting-state functional magnetic resonance imaging (rs-fMRI). It found altered functional connectivity in several brain regions, including the thalamus, frontal lobe, and parietal lobe, which are consistent with our findings of increased CBF in these regions after acupuncture treatment. This suggests that acupuncture may help restore normal neural activity in these key areas involved in sleep regulation.

Other studies have also reported similar results. For instance, research assessing local brain glucose metabolism in insomnia patients and healthy subjects found a small decline in relative glucose metabolism from sleep to wakefulness in brain regions associated with cognition and mood, such as the anterior cingulate cortex, hippocampus, amygdala, insula, and prefrontal cortex, as well as arousal regions like the thalamus, hypothalamus, and ascending reticular activating system (19). Our study’s finding of increased CBF in the thalamic nucleus and other relevant brain regions after acupuncture treatment aligns with the concept that enhancing neural activity in these areas can improve sleep and mood.

In the context of acupuncture research, previous studies have shown that acupuncture can modulate brain activity in a complex manner. It is not a simple one-to-one correspondence between acupuncture points and the central nervous system but involves a wide range of nervous systems (20). Our study further supports this by demonstrating that acupuncture at specific points can lead to increased CBF in multiple brain regions, which may contribute to its therapeutic effects on insomnia.

### Plausible mechanisms

From a neurophysiological perspective, the cerebellar cortex, traditionally known for its role in motor control, is now recognized to participate in sleep regulation (21). Increased CBF in the cerebellar cortex may enhance its neural activity, enabling it to better interact with brainstem sleep-regulating centers like the raphe nuclei and locus coeruleus. These centers produce neurotransmitters such as serotonin and norepinephrine, which are crucial for sleep regulation (22). By strengthening these interactions, acupuncture treatment may promote a more stable and regular sleep pattern (23).

The thalamus is central to sleep regulation, especially through the thalamocortical circuit. This circuit generates sleep spindles during non-rapid eye movement (NREM) sleep, which are essential for sleep consolidation and long-term memory storage by facilitating the transfer of information from the hippocampus to the neocortex (24). An increase in CBF in the thalamic nucleus implies enhanced activity in the thalamocortical circuit, leading to more efficient sleep spindle generation and improved NREM sleep quality (25). Moreover, the thalamus acts as a sensory information relay station. Increased CBF may enhance its filtering function, reducing the transmission of irrelevant sensory stimuli to the cortex during sleep, minimizing sleep interruptions, and improving overall sleep quality (26). Regarding anxiety relief, the thalamus processes emotional information, receiving inputs from limbic structures like the amygdala and relaying them to the prefrontal cortex (27). Increased CBF in the thalamic nucleus may enhance its ability to regulate emotional information flow, preventing amygdala over-activation, which is closely linked to anxiety, thus promoting a more balanced emotional state and reducing anxiety symptoms (28).

The increased CBF in the occipital, frontal, and parietal lobes may also contribute to the improvement of sleep quality and anxiety symptoms. The occipital lobe is mainly involved in visual processing, and its enhanced activity may help regulate the sleep- wake cycle by influencing the perception of light and darkness (29). The frontal lobe is crucial for cognitive functions, including attention, decision-making, and emotional regulation. Increased CBF in the frontal lobe may improve cognitive control over sleep-related thoughts and emotions, reducing anxiety and promoting better sleep (30). The parietal lobe is involved in sensory integration and spatial awareness, and its increased activity may help the brain better process sensory information during sleep, leading to a more restful sleep state (31).

### Clinical implications

The findings of our study have important clinical implications. The combined treatment of acupuncture at Baihui, Yintang, and Neiguan acupoint with conventional Western medicine offers a promising approach for the management of insomnia. It not only improves sleep quality but also alleviates anxiety symptoms, which are often comorbid with insomnia. This integrated treatment method may provide a more comprehensive and effective solution for patients compared to single-modality treatments.

In addition, the use of fMRI to visualize the effects of acupuncture on brain function adds a new dimension to the clinical application of acupuncture. It allows clinicians to better understand the underlying mechanisms of acupuncture’s therapeutic effects and may help in optimizing acupuncture treatment protocols. For example, by identifying the specific brain regions that respond to acupuncture, clinicians can target these areas more precisely during treatment, potentially enhancing the treatment’s efficacy.

### Limitations and future directions

Despite the promising results, our study has several limitations. First, the sample size was relatively small, which may limit the generalizability of the findings. Larger-scale studies are needed to confirm the effectiveness and reliability of the combined treatment in a more diverse population of insomnia patients.

Second, the 28-day treatment duration may be relatively short. Long-term effects of the combined treatment on sleep quality, anxiety symptoms, and brain function changes are not well-understood. A longer follow-up period would be beneficial to assess the sustainability of the treatment effects and potential long-term side effects.

Third, although we tried to control for some confounding factors, there may still be other unmeasured variables that could influence the results. For example, patients’ lifestyle factors such as diet, exercise habits, and stress levels were not comprehensively accounted for in the study. These factors could potentially interact with the treatment and affect the outcomes. Future studies should include a more comprehensive assessment of these confounding factors.

Fourth, in our study, it was challenging to achieve complete blinding of both patients and acupuncturists. Patients may have had preconceived notions about acupuncture treatment, and acupuncturists may have unconsciously influenced the treatment process. The lack of strict blinding could introduce bias into the results. Future studies should consider using sham acupuncture controls to better control for placebo and expectation effects, especially when evaluating subjective measures such as PSQI and HAMA.

Finally, our study did not prospectively register. We are aware that prospective registration is a recommended practice in research as it enhances transparency and reduces the risk of selective reporting. We acknowledge the limitation of not having prospective registration and will make sure to register our future studies prospectively to better align with research best practices.

## Conclusion

Acupuncture at Baihui, Yintang, and Neiguan acupoint combined with conventional Western medicine treatment shows great potential in improving the sleep quality and anxiety symptoms of insomnia patients. The fMRI findings provide valuable insights into the neural mechanisms underlying acupuncture’s effects. However, further research with larger sample sizes, longer treatment durations, better control of confounding factors, and strict blinding is needed to fully validate these findings and optimize the clinical application of this combined treatment approach.

## Data Availability

The raw data supporting the conclusions of this article will be made available by the authors, without undue reservation.
